# Home Blood Pressure Monitoring Among Adolescents and Young Adults, NHANES 2009–2014

**DOI:** 10.1093/ajh/hpaf069

**Published:** 2025-04-28

**Authors:** Rushelle L Byfield, Eunhee Choi, Jordana B Cohen, Ian M Kronish, Michael Rakotz, Daichi Shimbo

**Affiliations:** Division of Pediatric Nephrology and Hypertension, Department of Pediatrics, Columbia University Vagelos College of Physicians and Surgeons, New York, NY, USA; Columbia Hypertension Laboratory, Department of Medicine, Columbia University Irving Medical Center, New York, NY, USA; Renal-Electrolyte and Hypertension Division, Perelman School of Medicine, University of Pennsylvania, Philadelphia, PA, USA; Center for Behavioral Cardiovascular Health, Columbia University Irving Medical Center, New York, NY, USA; American Medical Association, Chicago, IL, USA; Columbia Hypertension Laboratory, Department of Medicine, Columbia University Irving Medical Center, New York, NY, USA

**Keywords:** adolescent, blood pressure, home blood pressure monitoring, hypertension, young adult

## Abstract

**BACKGROUND:**

Among middle-aged and older adults, home blood pressure (BP) monitoring (HBPM) is a tool for both diagnosis and management of hypertension. Pediatric guidelines recommend HBPM as an adjunct to office BP measurement among children and adolescents with hypertension. Little is known about the prevalence and frequency of HBPM in the United States among youth with hypertension, and what factors are associated with HBPM use.

**METHODS:**

We examined data from NHANES (National Health and Nutrition Examination Survey) 2009–2014 cycles to determine HBPM use in adolescents (16 to <20 years) and young adults (20 to <25 years), *n* = 2,919. HBPM frequency was defined as none, less than once a month, or at least monthly. Using multivariable logistic regression, we assessed the association between HBPM and sociodemographic factors including age, sex, race/ethnicity, obesity, income, insurance status, and education.

**RESULTS:**

An estimated 10.3% of adolescents and young adults reported performing HBPM in the past year. Of those with hypertension, 22.8% reported performing any HBPM in the past year. Compared to adolescents, a higher percentage of young adults reported performing HBPM (12.5% vs. 7.3%). In adjusted models, having health insurance (odds ratio [OR] 1.84 [95% confidence interval {CI} 1.25–2.73]), high school education or greater (OR 1.79 [95% CI 1.13–2.82]), and obesity (OR 1.83 [95% CI 1.10–3.05]) were associated with reporting any HBPM use.

**CONCLUSIONS:**

In this analysis of nationally representative US data, HBPM was reported in less than a quarter of adolescents and young adults with hypertension. Obesity, insurance status, and education were associated with any HBPM use.

## Graphical Abstract

**Graphical Abstract:**
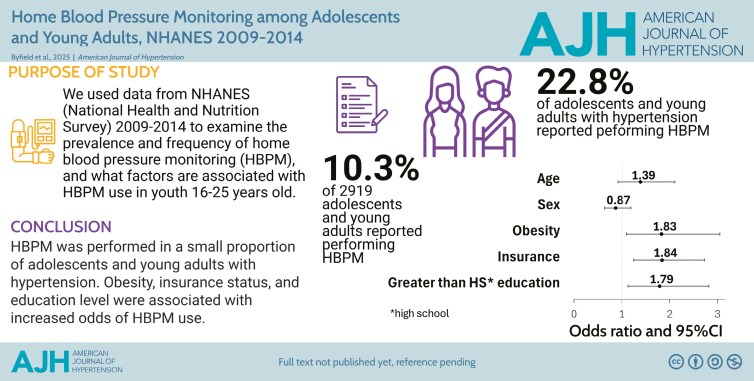

Home blood pressure (BP) monitoring (HBPM), the measurement of BP in the home setting, is a valuable tool for the diagnosis and management of hypertension in the adult population.^1^ Youth with hypertension are susceptible to delayed diagnosis and treatment of their hypertension^2,3^ as well as poor BP control.^4^ The 2017 pediatric hypertension clinical practice guidelines recommend HBPM as an adjunct to office BP measurement among children and adolescents once hypertension has been diagnosed,^5^ and HBPM may also be useful for the initial diagnosis of hypertension.^6^ Since adolescence and early young adulthood represent a period where lifestyle behaviors and cardiovascular risk factors develop, interventions during this period can contribute to improved long-term cardiovascular health. Although previous studies have investigated HBPM prevalence and frequency, data for adolescents aged 16–18 years were not examined, and older adolescents and young adults (≥18 years) were grouped together with those in later adulthood.^7,8^ We used data from the 2009 to 2014 NHANES (National Health and Nutrition Examination Survey) cycles to examine the prevalence and frequency of self-reported HBPM, and sociodemographic factors associated with HBPM use among US adolescents (ages 16 to <20 years) and young adults (ages 20 to <25 years).

## METHODS

### Survey description

NHANES is a series of cross-sectional studies performed in 2-year cycles conducted by the National Center for Health Statistics (NCHS), a part of the Centers for Disease Control and Prevention. Standardized health interviews and physical examinations were performed. Details of the NHANES sampling design, interview, and examination protocol have been described elsewhere.^9^ The study protocol for each NHANES cycle was approved by the NCHS institutional review board.

### Study sample

This analysis focused on the 2009–2014 NHANES cycles during which an HBPM use questionnaire was administered. A total of 30,468 participants were sampled. Of those, 3,574 (11.7%) were aged 16–25 years. Participants were included if they completed the health interview and medical examination, had complete data on the HBPM questionnaire, were nonpregnant, and had complete data on the covariates of interest. The final analysis sample included 2,919 participants (Figure S1).

### HBPM use and frequency

Survey respondents were asked^10^: “Did you take your BP at home during the last 12 months?” Those that answered yes were asked: “How often did you check your BP at home during the last 12 months?” HBPM frequency was recorded by the number of times per year (on average) and categorized into three mutually exclusive groups. Those that answered no were classified as HBPM frequency of “none.” An answer of performing HBPM an average of 1–11 times in the past 12 months was categorized as less than once a month; an average of 12 or more was categorized at least once a month.^7^

### Exam BP measurement and hypertension status

Trained physicians obtained three consecutive BP readings using the auscultatory method with a mercury manometer.^11^ Among those ≥18 years old, hypertension status was defined as having a self-reported history of hypertension or measured mean systolic BP (SBP) ≥140 mmHg or mean diastolic BP (DBP) ≥90 mmHg.^12^ For those <18 years old, hypertension was defined as having self-reported history of hypertension, or measured SBP or DBP ≥95th percentile based on age, sex, and height.^13^ We elected to use BP thresholds from guidelines that were contemporary to when the NHANES study was performed.^12,13^ Notably, BP readings were taken at a single visit, not multiple separate occasions, and therefore do not meet guideline criteria for a formal diagnosis of hypertension.^12,13^

### Covariates

Race and ethnicity were self-reported and classified as non-Hispanic White, non-Hispanic Black, Hispanic, and Others which included those who identified as multiracial. Age groups were classified as 16 to <20 years old and ≥20 to <25 years old. Income was determined using the family income-to-poverty ratio (IPR) which is calculated by the ratio of household income to poverty threshold as defined by the US Department of Health and Human Services for each specific survey year.^14^ Insurance status was determined by answer of yes to the interview question: “Are you covered by health insurance or some other kind of healthcare plan.” Education level was categorized as: 12th grade or less vs. high school graduate/general education diploma or greater. Lastly, body mass index (BMI) was determined by dividing weight in kilograms by measured height in meters squared. Obesity was defined by BMI ≥30 kg/m^2^ in those aged ≥20 or BMI ≥95th percentile for age and sex in those aged <20 years.^15^

### Statistical analysis

Baseline characteristics of the 2,919 participants were determined and stratified by age group. All analyses used sampling weights to account for the complex survey design including oversampling, and survey nonresponse. Analyses were also post-stratified to obtain nationally representative estimates of the civilian, noninstitutionalized US population. The frequency of HBPM (none, less than once a month, and at least once a month) was determined in the overall sample, and among subgroups defined by age group, sex, race and ethnicity, and hypertension status. NCHS-recommended presentation standards for proportions were applied.^16^ The percentages of those with hypertension with HBPM frequency less than once a month and at least once a month were suppressed due to NCHS recommendations.^16^ Logistic regression was used to examine associations of specific factors with any HBPM use in the prior 12 months. Univariate and adjusted models were examined. The adjusted model included age, sex, race and ethnicity, obesity, hypertension status, family IPR, insurance status, and education level. A P-value <0.05 was considered to be statistically significant. All analyses were performed using SAS Version 9.4 (SAS Institute, Cary, NC).

## RESULTS

### Baseline characteristics

Among US adolescents and young adults aged 16 to <25 years, 47.5% were female, 57.2% were Non-Hispanic White, 14.5% were Non-Hispanic Black, and 19.7% were Hispanic. The mean (95% confidence interval [CI]) age was 17.4 (17.3–17.5) years in the 16 to <20 years age group, and 21.9 (21.8–22.1) years in the 20 to <25 years age group (Table S1). Hypertension was present in 2.7% of the 16 to <20 years age group and 3.6% of the 20 to <25 years age group. Among those in the analytic cohort classified with hypertension, 23.8% had hypertension defined by self-report alone, 70.6% based on exam-measured BP alone, and 5.6% had both.

### Prevalence and frequency of HBPM

In the overall group, 10.3% reported performing any HBPM in the previous 12 months: 6.6% less than once a month and 3.7% at least once a month (Table 1). There was a higher report of any HBPM in participants aged 20 to <25 years compared to those aged 16 to <20 years (12.5% vs. 7.3%, *P* < 0.0001). There was also a higher report of any HBPM in those with hypertension compared to those without hypertension (22.8% vs. 9.9%, *P* = 0.005). There were no statistically significant differences in the frequency of HBPM across subgroups defined by sex or race and ethnicity (Table 1).

**Table 1.** Frequency of home blood pressure monitoring in the previous 12 months.

	*N*	Frequency of HBPM, %				
		None	Any	Less than once a month	At least once a month	*P*-value^*^
Overall group	2,919	89.7%	10.3%	6.6%	3.7%	-
Age groups						
16 to <20 years	1,614	92.7%	7.3%	5.4%	1.9%	<0.0001
20 to <25 years	1,305	87.5%	12.5%	7.5%	5.0%	
Sex groups						
Male	1,519	89.3%	10.7%	7.6%	3.2%	0.5
Female	1,400	90.2%	9.8%	5.6%	4.2%	
Race and ethnicity groups						
Non-Hispanic White	942	88.6%	11.4%	7.8%	3.6%	0.1
Non-Hispanic Black	73	90.0%	10%	6.1%	3.9%	
Hispanic	839	92.5%	7.5%	3.6%	3.9%	
Others	399	89.9%	10.1%	6.7%	^**^	
Hypertension status^†^						
Without hypertension	2,830	90.1%	9.9%	6.4%	3.4%	0.005
With hypertension	89	77.2%	22.8%	^**^	^**^	

Abbreviation: BP, blood pressure; HBPM, home blood pressure monitoring.

^*^ *P*-value represents relationship between none vs. any HBPM across subgroups.

^**^These estimates may be unreliable due to small cell size and were suppressed according to NCHS standards.^11^

^†^Hypertension is defined as self-reported history of hypertension or measured systolic BP ≥ 140 mmHg or diastolic BP ≥ 90 mmHg in those ≥18 years old or measured systolic or diastolic BP ≥ 95th percentile for age/sex/height in those <18 years old.

All analyses used sampling weights to account for the complex survey design (including oversampling), survey nonresponse, and are also post-stratified to obtain nationally representative estimates of the civilian, noninstitutionalized US population. Percentages may not total 100% due to rounding.

### Factors associated with any HBPM use

In univariate analysis, age 20 to < 25 years (odds ratio [OR] 1.82, 95% CI 1.28–2.57), obesity (OR 1.86, 95% CI 1.12–3.09), hypertension (OR 2.70, 95% CI 1.30–5.63), having insurance (OR 1.68, 95% CI 1.16–2.42) and higher education level (high school graduate/GED or greater [OR 2.13, 95% CI 1.43–3.18]) were associated with higher odds of reporting any HBPM use in the prior 12 months (Table S2). However, race and ethnicity, sex, and IPR were not associated with reporting HBPM use. In an adjusted model including age, sex, race/ethnicity, obesity, hypertension status, IPR, insurance status, and education level, obesity (aOR [adjusted odds ratio] 1.83, 95% CI 1.10–3.05), having insurance (aOR 1.84, 95% CI 1.25–2.73) and higher education level (aOR 1.79, 95% CI 1.13–2.82) were associated with higher odds of reporting any HBPM in the prior 12 months. Hypertension was also associated with higher odds of reporting HBPM use (aOR 2.14, 95% CI 0.99–4.63).

## DISCUSSION

In this analysis, we found the majority of adolescents and young adults did not perform HBPM, including those with hypertension. After multivariable adjustment for sociodemographic and clinical characteristics, factors that were independently associated with HBPM use were hypertension status, obesity, health insurance, and higher education level. Our findings draw attention to the low uptake of HBPM in adolescents and young adults with hypertension, and the potential role of obesity and socioeconomic status in HBPM use.

Using NHANES 2011–2014 data, Ostchega *et al.* found that 48% of US adults ≥18 years with hypertension reported performing HBPM.^8^ In our study, using NHANES 2009–2014 data, more than three-quarters of adolescents and young adults with hypertension reported no HBPM use in the previous 12 months. Possible explanations for the low use of HBPM may be youth lacking awareness of their hypertension status,^17^ or the patient or their clinician did not perceive HBPM as an important part of hypertension management^8^ as there was little to no mention of HBPM in contemporary US hypertension guidelines.^12,13^ The most recent 2017 pediatric hypertension guideline supports HBPM as an adjunct for hypertension management^5^; however, NHANES has not collected additional data on HBPM use in adolescents and young adults since 2014.

Healthcare engagement decreases in the adolescent and young adult age groups. Care gaps of approximately 21 months for office visits and 42 months for preventive visits were noted during the transition from pediatric to adult care.^18^ These healthcare gaps are not trivial as longer time between clinic visits are associated with worse BP control in young adults.^4^ Given uncontrolled BP carries an increased risk for adverse cardiovascular events,^19^ promoting BP control in this population represents an opportunity to improve cardiovascular health. Although the 2017 pediatric hypertension guidelines only recommend HBPM for hypertension management, HBPM can potentially play a role in hypertension screening as well. In the current study, approximately 10% of those without hypertension (i.e., not having high office BP at the NHANES visit, and did not report a history of hypertension) reported performing HBPM. These participants may have used an HBPM device to check whether they developed hypertension without HBPM being recommended by a clinician. Alternatively, it is possible that these participants had high BP at a clinic visit but not the NHANES visit and were instructed by a clinician to perform BP measurements at home. These scenarios highlight the potential use of HBPM for the initial diagnosis of hypertension. For hypertension management, several studies in adults have shown that HBPM can contribute to enhancing BP control.^20^ The opportunity to combine HBPM with telemonitoring makes it ideal for engaging youth with their BP management and decreases the need for in-person office visits. More work needs to be done to develop pediatric-specific HBPM normative data and validated BP devices with appropriately sized cuffs for youth.

This study has several strengths including the use of a large nationally representative sample with well-described anthropometric and sociodemographic characteristics and office BP measurements that were obtained by trained staff using a standardized methodology. There are also potential limitations. HBPM was determined by self-report, which could be susceptible to recall bias. Further, parental reports were not available to confirm HBPM use in adolescents. Hypertension was defined by office BP measurements at the NHANES visit or a self-report of a history of hypertension; therefore, misclassification of hypertension is possible. Small numbers of adolescents and young adults with hypertension limited statistical power and some estimates needed to be suppressed. Given small sample sizes of some HBPM frequency categories, we did not have sufficient power to perform analyses on more granular HBPM frequencies. With regards to education level, it should be noted that in our analysis cohort, only 3% of 16- to 17-year olds had completed high school; therefore, the interpretation of the association of education with HBPM in the adolescent age group must be made with caution.

In conclusion, the performance of HBPM was low among adolescents and youth with hypertension. Obesity, having health insurance, and higher education were associated with HBPM use. These findings suggest there may be a missed opportunity for incorporating HBPM to optimize hypertension screening, diagnosis, and management. The transition from pediatric to adult care is a vulnerable period where HBPM could play a particularly helpful role in engaging and managing those with high cardiovascular disease risk.

## Supplementary Data

Supplementary materials are available at *American Journal of Hypertension* (http://ajh.oxfordjournals.org).

hpaf069_suppl_Supplementary_Figure_S1_Tables_S1-S2

## Data Availibility

The datasets were derived from sources in the public domain: https://wwwn.cdc.gov/nchs/nhanes/continuousnhanes/default.aspx?BeginYear=2009, https://wwwn.cdc.gov/nchs/nhanes/continuousnhanes/default.aspx?BeginYear=2011, and https://wwwn.cdc.gov/nchs/nhanes/continuousnhanes/default.aspx?BeginYear=2013.

## References

[1] Whelton PK, Carey RM, Aronow WS, Casey DE Jr, Collins KJ, Dennison Himmelfarb C, DePalma SM, Gidding S, Jamerson KA, Jones DW, MacLaughlin EJ, Muntner P, Ovbiagele B, Smith SC Jr, Spencer CC, Stafford RS, Taler SJ, Thomas RJ, Williams KA Sr, Williamson JD, Wright JT Jr. 2017 ACC/AHA/AAPA/ABC/ACPM/AGS/APhA/ASH/ASPC/NMA/PCNA guideline for the prevention, detection, evaluation, and management of high blood pressure in adults: executive summary: a report of the American College of Cardiology/American Heart Association Task Force on Clinical Practice Guidelines. Hypertension 2018; 71:1269–1324. 29133354 10.1161/HYP.0000000000000066

[2] Johnson HM, Thorpe CT, Bartels CM, Schumacher JR, Palta M, Pandhi N, Sheehy AM, Smith MA. Undiagnosed hypertension among young adults with regular primary care use. J Hypertens 2014; 32:65–74. 24126711 10.1097/HJH.0000000000000008 PMC3868024

[3] Johnson HM, Thorpe CT, Bartels CM, Schumacher JR, Palta M, Pandhi N, Sheehy AM, Smith MA. Antihypertensive medication initiation among young adults with regular primary care use. J Gen Intern Med 2014; 29:723–731. 24493322 10.1007/s11606-014-2790-4 PMC4000352

[4] King CC, Bartels CM, Magnan EM, Fink JT, Smith MA, Johnson HM. The importance of frequent return visits and hypertension control among US young adults: a multidisciplinary group practice observational study. J Clin Hypertens (Greenwich) 2017; 19:1288–1297. 28929608 10.1111/jch.13096 PMC5722664

[5] Flynn JT, Kaelber DC, Baker-Smith CM, Blowey D, Carroll AE, Daniels SR, de Ferranti SD, Dionne JM, Falkner B, Flinn SK, Gidding SS, Goodwin C, Leu MG, Powers ME, Rea C, Samuels J, Simasek M, Thaker VV, Urbina EM; Subcommittee on Screening and Management of High Blood Pressure in Children. Clinical practice guideline for screening and management of high blood pressure in children and adolescents. Pediatrics 2017; 140:e20171904. 28827377 10.1542/peds.2017-1904

[6] Stergiou G, Stambolliu E, Bountzona I, Ntineri A, Kollias A, Vazeou A, Soldatou A. Home blood pressure monitoring in children and adolescents: systematic review of evidence on clinical utility. Curr Hypertens Rep 2019; 21:64. 31240404 10.1007/s11906-019-0967-2

[7] Ostchega Y, Berman L, Hughes JP, Chen TC, Chiappa MM. Home blood pressure monitoring and hypertension status among US adults: the National Health and Nutrition Examination Survey (NHANES), 2009-2010. Am J Hypertens 2013; 26:1086–1092. 23604493 10.1093/ajh/hpt054

[8] Ostchega Y, Zhang G, Kit BK, Nwankwo T. Factors associated with home blood pressure monitoring among US adults: National Health and Nutrition Examination Survey, 2011-2014. Am J Hypertens 2017; 30:1126–1132. 28633432 10.1093/ajh/hpx101 PMC9880871

[9] Curtin LR, Mohadjer LK, Dohrmann SM, Kruszon-Moran D, Mirel LB, Carroll MD, Hirsch R, Burt VL, Johnson CL. National Health and Nutrition Examination Survey: sample design, 2007-2010. Vital Health Stat 2013;2:1–23. 25090039

[10] 2009 English SP Questionnaire–Blood Pressure <https://wwwn.cdc.gov/nchs/data/nhanes/public/2009/questionnaires/bpq_f.pdf>

[11] National Health and Nutrition Examination Survey (NHANES) Health Tech/Blood Pressure Procedures Manual 2009 < https://wwwn.cdc.gov/nchs/data/nhanes/public/2009/manuals/BP.pdf>.

[12] Chobanian AV, Bakris GL, Black HR, Cushman WC, Green LA, Izzo JL Jr, Jones DW, Materson BJ, Oparil S, Wright JT Jr, Roccella EJ; National Heart, Lung, and Blood Institute Joint National Committee on Prevention, Detection, Evaluation, and Treatment of High Blood Pressure. The seventh report of the Joint National Committee on Prevention, Detection, Evaluation, and Treatment of High Blood Pressure: the JNC 7 report. JAMA 2003; 289:2560–2572. 12748199 10.1001/jama.289.19.2560

[13] National High Blood Pressure Education Program Working Group on High Blood Pressure in C, Adolescents. The fourth report on the diagnosis, evaluation, and treatment of high blood pressure in children and adolescents. Pediatrics 2004; 114:555–576. 15286277

[14] Services UDoHaH. Annual Update of the HHS Poverty Guidelines 2011 <https://www.federalregister.gov/d/2011-1237>

[15] Hampl SE, Hassink SG, Skinner AC, Armstrong SC, Barlow SE, Bolling CF, Avila Edwards KC, Eneli I, Hamre R, Joseph MM, Lunsford D, Mendonca E, Michalsky MP, Mirza N, Ochoa ER, Sharifi M, Staiano AE, Weedn AE, Flinn SK, Lindros J, Okechukwu K. Clinical practice guideline for the evaluation and treatment of children and adolescents with obesity. Pediatrics 2023; 151:e2022060641. 36622115 10.1542/peds.2022-060640

[16] Parker JD, Talih M, Malec DJ, Beresovsky V, Carroll M, Gonzalez JF, Hamilton BE, Ingram DD, Kochanek K, McCarty F, Moriarity C, Shimizu I, Strashny A, Ward BW. National Center for Health Statistics Data presentation standards for proportions. Vital Health Stat 2017;2:1–22. 30248016

[17] Gooding HC, McGinty S, Richmond TK, Gillman MW, Field AE. Hypertension awareness and control among young adults in the National Longitudinal Study of Adolescent Health. J Gen Intern Med 2014; 29:1098–1104. 24577758 10.1007/s11606-014-2809-x PMC4099443

[18] Wisk LE, Finkelstein JA, Sawicki GS, Lakoma M, Toomey SL, Schuster MA, Galbraith AA. Predictors of timing of transfer from pediatric- to adult-focused primary care. JAMA Pediatr 2015; 169:e150951. 26030515 10.1001/jamapediatrics.2015.0951 PMC4862601

[19] Zhou D, Xi B, Zhao M, Wang L, Veeranki SP. Uncontrolled hypertension increases risk of all-cause and cardiovascular disease mortality in US adults: the NHANES III Linked Mortality Study. Sci Rep 2018; 8:9418. 29925884 10.1038/s41598-018-27377-2 PMC6010458

[20] Agarwal R, Bills JE, Hecht TJ, Light RP. Role of home blood pressure monitoring in overcoming therapeutic inertia and improving hypertension control: a systematic review and meta-analysis. Hypertension 2011; 57:29–38. 21115879 10.1161/HYPERTENSIONAHA.110.160911

