# Supplementary material for: Home Blood Pressure Monitoring Among Adolescents and Young Adults, NHANES 2009–2014
Source: Am J Hypertens. 2025 Apr 28;38(10):754–8. doi: 10.1093/ajh/hpaf069 (PMC12550363; doi:10.1093/ajh/hpaf069)
Supplement: hpaf069_suppl_Supplementary_Figure_S1_Tables_S1-S2 [file hpaf069_suppl_supplementary_figure_s1_tables_s1-s2.docx]

**Home blood pressure monitoring among adolescents and young adults, NHANES 2009-2014**

Rushelle L. Byfield, Eunhee Choi, Jordana B. Cohen, Ian M. Kronish, Michael Rakotz, Daichi Shimbo

**Supplemental Materials**

1. **Table S1** Baseline Characteristics by Age Group (weighted frequencies)
2. **Table S2** Factors associated with having reported any HBPM use in the prior 12 months
3. **Figure S1** Cohort Derivation

**Table S1. Baseline Characteristics by Age Group (weighted frequencies)**

|  | Overall | Age group, % (95% CI) | |
| --- | --- | --- | --- |
|  | N = 2919 | 16 to <20  (N=1614) | 20 to <25  (N=1305) |
| Age mean (95% CI) | 20.0 (19.8 - 20.2) | 17.4 (17.3 – 17.5) | 21.9 (21.8 – 22.1) |
| Female sex, % | 47.5% | 47.1% | 47.8% |
| Race/ethnicity, %  Non-Hispanic white  Non-Hispanic black  Hispanic  Others | 57.2%  14.5%  19.7%  8.6% | 56.1%  15.1%  20.4%  8.3% | 58.0%  14.1%  19.1%  8.8% |
| Obesity*, % | 22.6% | 19.7% | 24.7% |
| Hypertension status**, % | 3.3% | 2.7% | 3.6% |
| Income to poverty Ratio  <1.3  1.3 -3.49  ≥3.5 | 40.1%  34.4%  25.5% | 35.7%  34.8%  29.6% | 43.3%  34.2%  22.5% |
| Has insurance, % | 76.0% | 85.4% | 69.0% |
| Education level, %  12th grade or less  High school grad/GED or greater | 37.0%  63.0% | 68.2%  31.8% | 14.0%  86.0% |

Data are presented as mean (95% CI) or %

Abbreviations: BP = blood pressure, CI= confidence interval, eGFR = estimated glomerular filtration fraction, GED = general education diploma

Percentages represent weighted frequencies.

*Obesity is defined by BMI ≥30kg/m^2^ in those age ≥20 or BMI ≥95^th^ percentile in those age <20. **Hypertension is defined as self-reported history of hypertension or measured systolic BP ≥140 mmHg or diastolic BP ≥90 mmHg in those ≥18 years old or measured systolic or diastolic BP ≥95^th^ percentile for age/sex/height in those <18 years old.

**Table S2. Factors associated with having reported any HBPM use in the prior 12 months**

|  | Univariate | Adjusted Model |
| --- | --- | --- |
| Factors | OR 95%CI | OR (95% CI) |
| Age  16 to < 20 years  20 to < 25 years | Ref  **1.82 (1.28 – 2.57)** | Ref  1.39 (0.92 – 2.11) |
| Sex  Female  Male | 0.90 (0.67 – 1.22)  Ref | 0.87(0.64 – 1.19)  Ref |
| Race/ethnicity  Non-Hispanic white  Non-Hispanic black  Hispanic  Others | 1.14 (0.75 – 1.74)  0.99 (0.60 – 1.63)  0.72 (0.43 – 1.21)  Ref | 1.06 (0.70 – 1.59)  0.91 (0.54 – 1.54)  0.80 (0.64 – 1.19)  Ref |
| Obesity* (yes/no) | **1.86 (1.12 – 3.09)** | **1.83 (1.10 – 3.05)** |
| Hypertension status** (yes/no) | **2.70 (1.30 – 5.63)** | 2.14 (0.99 – 4.63) |
| Income to poverty Ratio  <1.3  1.3 -3.49  ≥3.5 | Ref   1. (0.55 – 1.80)   1.07(0.60 – 1.91) | Ref  0.94 (0.53 – 1.67)  0.87 (0.50 – 1.54) |
| Has insurance (yes/no) | **1.68 (1.16 – 2.42)** | **1.84 (1.25 – 2.73)** |
| Education level  12^th^ grade or less  High school grad/GED  or greater | Ref  **2.13 (1.43 – 3.18)** | Ref  **1.79 (1.13 – 2.82)** |

Abbreviations: CI= confidence interval, GED = general education diploma, HBPM = home blood pressure monitoring, OR = odds ratio

Adjusted model includes: age, sex, race/ethnicity, obesity, hypertension status, income to poverty ratio, insurance status, and education level

*Obesity is defined by BMI ≥30kg/m^2^ in those age ≥20 or BMI ≥95^th^ percentile in those age <20.

**Hypertension is defined as self-reported history of hypertension or measured systolic BP ≥140 mmHg or diastolic BP ≥90 mmHg in those ≥18 years old or measured systolic or diastolic BP ≥95th percentile for age/sex/height in those <18 years old

**Figure S1****. Cohort Derivation**

Participants sampled in 2009-2014 NHANES

N= 30468

­­

Excluded due to Age <16 or >25 years

N=26894

**Final Cohort**

**N=2919**

Age 16-25 years

N=3574

Excluded due to pregnancy or missing data on: HBPM questionnaire, clinic blood pressure measurement, sex, height, body mass index, income, education level, insurance status

N=655

Abbreviations: HBPM=home blood pressure monitoring, NHANES= National Health and Nutrition Examination Survey
